# EDLaaS:Fully Homomorphic Encryption over Neural Network Graphs for Vision and Private Strawberry Yield Forecasting

**DOI:** 10.3390/s22218124

**Published:** 2022-10-24

**Authors:** George Onoufriou, Marc Hanheide, Georgios Leontidis

**Affiliations:** 1 School of Computer Science, University of Lincoln, Lincoln LN6 7TS, UK; 2 Interdisciplinary Centre for Data and AI & School of Natural and Computing Sciences, University of Aberdeen, Aberdeen AB24 3FX, UK

**Keywords:** fully homomorphic encryption, deep learning, machine learning, privacy-preserving technologies, agri-food, data sharing

## Abstract

We present automatically parameterised Fully Homomorphic Encryption (FHE) for encrypted neural network inference and exemplify our inference over FHE-compatible neural networks with our own open-source framework and reproducible examples. We use the fourth generation Cheon, Kim, Kim, and Song (CKKS) FHE scheme over fixed points provided by the Microsoft Simple Encrypted Arithmetic Library (MS-SEAL). We significantly enhance the usability and applicability of FHE in deep learning contexts, with a focus on the constituent graphs, traversal, and optimisation. We find that FHE is not a panacea for all privacy-preserving machine learning (PPML) problems and that certain limitations still remain, such as model training. However, we also find that in certain contexts FHE is well-suited for computing completely private predictions with neural networks. The ability to privately compute sensitive problems more easily while lowering the barriers to entry can allow otherwise too-sensitive fields to begin advantaging themselves of performant third-party neural networks. Lastly, we show how encrypted deep learning can be applied to a sensitive real-world problem in agri-food, i.e., strawberry yield forecasting, demonstrating competitive performance. We argue that the adoption of encrypted deep learning methods at scale could allow for a greater adoption of deep learning methodologies where privacy concerns exist, hence having a large positive potential impact within the agri-food sector and its journey to net zero.

## 1. Introduction

**Figure 1:**
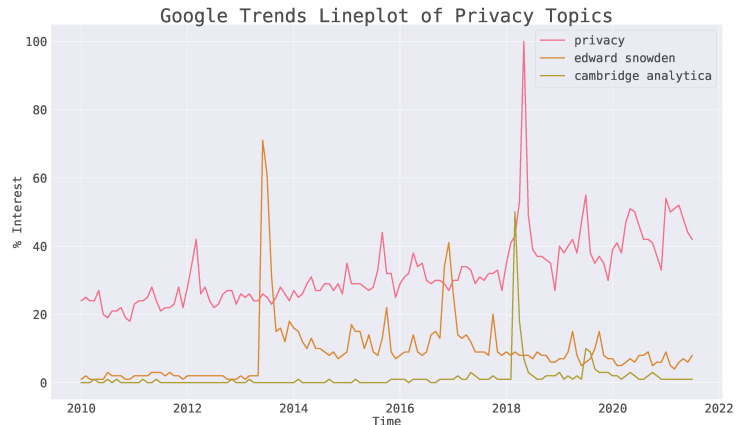
Trends of privacy (red), Edward Snowden (orange), and Cambridge Analytica topics (green) on Google trends since 2010 showing a slow but steady increase in the interest in privacy and particular peaks around events such as the Cambridge Analytica scandal and smaller peaks roughly correlated to Julian Assange [3].

Privacy is slowly becoming of greater interest (Figure 1) to the broader public, especially during and after particular scandals, such as Cambridge Analytica (corporate actors), Edward Snowden on the five eyes (state actors) [1], and more recently the Pegasus project on the cyberarms NSO group (both corporate and state). This increased concern for privacy has over time manifested itself in many forms; one of the most notable examples being in legislation such as the General Data Protection Regulation (GDPR) [2].

A less thought-of field where privacy is of concern is the agri-food sector. Agriculturalists often are incredibly reluctant to share data, due to real, or perceived sensitivity. We believe that this data sharing reluctance originates from two factors; data are not being collected due to the unawareness of the value-for-cost they can offer, and data are not shared due to concerns over loss of competitiveness if their techniques were leaked. This means it is incredibly difficult for new and possibly disruptive approaches to be used for forecasting and thus later optimising some component in the agri-food chain. One such disruptive approach is the application of deep learning which has become state of the art in almost all areas where sufficient data are present with which to train it. There are many reasons why such new approaches are necessary, but the key area we gear our work towards is tackling food waste at production, by forecasting accurate yields.

Here in the UK we have dual problems of food insecurity and high food waste. It is estimated that the annual combined surplus and food waste in primary production is 3.6 million tonnes (Mt) or 6–7% of total harvest. A further 9.5 Mt is wasted post-production/farm. A total of 7.7 Mt is wasted in-house, and 1.8 Mt is wasted in manufacturing and retail, while the total food purchased for consumption in the UK is 43 Mt [4]. More specifically, in the soft and stone fruit industry a large consortium of growers in 2018 overestimated by 17.7% for half of the growing season, while the remainder of the season they underestimated by 10%. Underestimation leads to surpluses which create extra cost in fruit disposal along with devaluing expected produce. Overestimation leads to fix-purchasing which entails importing fruit to cover the shortfall in the expected produce. This costs the consortium GBP 8 Million a year in losses, while the rest of the industry is estimated to have incurred GBP 18 Million losses a year at the time. The effect of climate change has been exacerbating the difficulties in yield forecasting due to the more erratic environmental conditions. Considering that freely available agri-food data are hard to find, given they are highly sensitive, progress in adopting AI technologies are hindered.

As far as using machine learning is concerned, it is extremely difficult to build and deploy neural network models to forecast agricultural yields due to the aforementioned privacy/sensitivity concerns that mean data for training and using these neural networks are scarce. However, the impact of using machine learning technologies in agri-food supply chains has been shown to be substantial [5,6,7]. A solution that involved distributed learning was recently proposed with an application on soy bean yield forecasting [8], which assumes that distributed training is possible. To provide an alternative solution to this, we propose to use fully homomorphic encryption (FHE) and demonstrate how it works and performs in a bespoke strawberry dataset (Katerina and Zara varieties) that was collected in our strawberry research facility in Riseholme Campus at the University of Lincoln, UK.

FHE affords us the ability to compute cyphertexts without the ability to detect or discern its contents, acting as a truly blind data processor in Encrypted Deep Learning as a Service (EDLaaS) applications [9]. In particular, EDLaaS is especially useful in highly sensitive/highly regulated industries such as medicine/patient data (especially due to GDPR), trade secrets, and military applications. FHE is not a panacea. Special care must be taken to ensure/maximise the security of cyphertexts, and the biggest problem with this is it is not immediately apparent if this is not ensured, often requiring a deep understanding of the underlying cryptography such that the parameterisation can be understood, analysed, and balanced against. However, a standard metric used thought of as a commonality is the number of bits used for the private keys. It is commonly considered that a private key with 128 bits is considered secure [10,11]. We maintain this minimum level of security throughout all our experimentation and implementations.

### Contributions

Our contributions towards FHE deep learning, given the current state of the field and related works (Section 2.2) are:

- Propose a new block-level automatic cyphertext parameterisation algorithm, which we call autoFHE. We also seek to showcase autoFHE in both regression and classification networks, which still appears to be a misunderstood and ongoing problem [12].
- Provide and showcase open-source encrypted deep learning with a reproducible step-by-step example on an open dataset, in this case Fashion-MNIST, achieved through a dockerised Jupyter-lab container, such that others can readily and easily explore FHE with deep learning and verify our results.
- Show a new application for encrypted deep learning to a confidential real-world dataset.
- Demonstrate how neuronal firing in multi-directed graphs can be achieved in our different approach.
- Show and detail precisely the computational graph of how a CNN can be constructed using FHE, in particular, how handling of the sum-of-products can occur. This, along with our easily reproduced example, should help clarify many otherwise omitted details from previous works.
- Show recent advancements in FHE compatibility such as ReLU approximations in greater detail along with problems/considerations as part of a whole computational graph. We also backpropogate the dynamically approximate range of ReLU.

## 2. Literature and Related Works

### 2.1. FHE Background

FHE is a structure-preserving encryption transformation [13], proposed by Craig Gentry in 2009 [14], allowing computation on cyphertexts (ε(x)) directly (addition and multiplication) without the need for decryption. This is what could be considered the first generation of FHE as implemented by Gentry in 2011 [15] and the Smart-Vercauteren implementation [16]. Gentry’s implementation for any given bootstrapping operating took anywhere from 30 s, for the smallest most “toy” example, to 30 min for the largest most secure example, with the former having a public key of 70 Megabytes, and the latter a public key of 2.4 Gigabytes in size [15]. Clearly this would be far too lengthy to be practically viable; however, there have been several generations of FHE since building on these initial works, improving computational and spacial complexity: second generation: BV [17], BGV [18], LTV [19], BFV [20], and BLLN [21]; third generation: GSW [22]; fourth generation: CKKS [23]. Here we focus on the Cheon, Kim, Kim, and Song (CKKS) scheme, for a plethora of reasons:

- Operates with fixed point precision unlike all other schemes, which is necessary for computation of neural networks with activations and inputs usually falling in the range 0,±1 [24]
- Has multiple available implementations (PALISADE [25], HEAAN [23], Microsoft Simple Encrypted Arithmetic Library (MS-SEAL) [10], HElib [26], etc). Only PALISADE [25] and Lattigo [27] are known to implement CKKS with bootstrapping, although many others have these features road-mapped.

**Figure 2:**
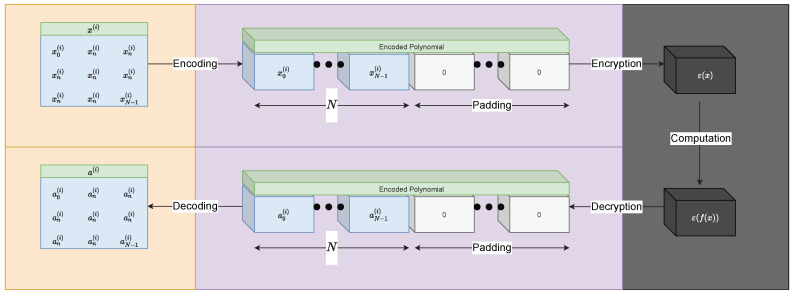
Fully homomorphic encryption (FHE) overview of distinct stages and properties [28].

Our implementation uses MS-Seal, a popular FHE library. Many of our techniques proposed here stretch to almost all other implementations since they follow the same basic rules, albeit with slightly different implications on things such as parameters. In this paper, we focus on using FHE without bootstrapping, or more precisely levelled fully homomorphic encryption (LFHE), meaning we calculate specific-sized although generalised (implementation) neural network circuits. Despite CKKS being the best candidate for forms of encrypted deep learning, it has certain shortcomings. Fundamentally, CKKS cyphertexts are the most atomic form of the data. This is a consequence of the optimisation used in many FHE schemes where a sequence of values (the “message” or plaintext data) are encoded into a single polynomial, and then this polynomial is what is then encrypted (Figure 2). This means there is less overhead since we are encrypting multiple values together, but it means we cannot operate on this value alone; we must always be homomorphic, i.e., maintain the same structure and operate on all values. Thus, if we encrypt a polynomial of length 10, that shall be the smallest form of the data until it is either bootstrapped or re-encrypted. Therefore, we are only able to operate on the 10 elements as a single whole, i.e., we cannot operate on the third element in the array alone to produce a single number answer. In addition, CKKS cyphertexts computational depth (pre-bootstrapping) is directly related to the length of the polynomial slots, which means we must choose our parameters carefully to ensure we do not have unnecessarily large cyphertexts and thus slow operations. Lastly, CKKS, as with many schemes, requires that two cyphertexts operating with each other must share the same parameters and be from the same private key. This means when, for instance, we have multiple inputs into a neural network, all directly interacting cyphertexts must be of the same key. This complicates some automatic parameterisation logic which we will discuss later.

### 2.2. Related Works

#### 2.2.1. Encrypted Deep Learning

There have been many other works that use FHE (bootstrappable) or Levelled FHE to compute some form of neural network. A few notable examples for FHE and convolutional neural networks (CNNs) are by Lee, [29], Meftah, [30], Juvekar [31], and Marcano, [32]. Lee uses a modified version of the Microsoft Simple Encrypted Arithmetic Library (MS-SEAL) to add bootstrapping as MS-SEAL does not currently support it. Lee shows FHE and DL used on the CIFAR-10 [33] dataset to mimic the ResNet-20 model achieving a classification accuracy of 90.67%. Juvekar uses the PALISADE library implementation of the BFV scheme with their own (LFHE) packed additive (PAHE) neural network framework to compute both MNIST and CIFAR-10. Meftah uses Homomorphic Encryption Library (HELib) [26] similar to Lee and is particularly focused on improving the practicality of (L) FHE as a means to compute a deep learning circuit. Meftah seeks to do this towards computing ImageNet [34] with the second generation BGV scheme [18] (on integers) as opposed to Lee using the fourth generation CKKS scheme [23] (on floating points). Lastly, Marcano previous is also concerned with the computational and spatial complexity of using FHE as a means to compute convolutional circuits. Marcano appears to use a custom FHE implementation on a fixed point number format, taking 36 hours to train on the MNIST dataset. It is unclear in all of these papers, however, exactly how the gradient descent or backward pass of the neural networks are implemented, which is necessary for neural network training. They also lack detail in key stages of the forward pass such as how they deal with calculating the sum of products of the CNN since a homomorphic cyphertext cannot be folded on itself to form a single number sum, or if they used point-wise encryption to be able to sum between cyphertexts or how they dealt with the sheer size of this plethora of cyphertexts. Lastly, the above papers do describe in some detail how some of their parameters are decided, in particular with regards to security, but they do not cover much on the computational depth or precision effects these parameters have on the cyphertext such as the modulus-switching chain.

#### 2.2.2. FHE Graph Parameterisation

Here, FHE graph parameterisation means deriving the FHE parameters from a graph, such as the computational depth and thus the parameters like the modulus size. There have been a few works that define FHE graph parameterisation, the most notable and similar of which is Microsoft Encrypted Vector Arithmetic (MS-EVA) [11,12]. MS-EVA uses Directed Acyclic Graphs (DAGs) to represent simple operations applied to some input constant. Since MS-EVA also uses MS-SEAL, this means it also uses RNS-CKKS, purportedly the most efficient CKKS implementation [11]. MS-EVA has been applied to encrypted deep learning inference, specifically LeNet-5 towards MNIST. Dathathri particularly emphasises the non-trivial nature and how parameterisation can be a large barrier to the adoption of FHE. However, there are no examples currently available to help lower this barrier. Subsequently, their nodes representing single atomic operations mean there is overhead when compared to block operations which could be an area of improvement.

### 2.3. Threat Model

Just like similar works in FHE, we assume a semi-honest/honest-but-curious threat model [11], where parties follow the specified protocol but attempt to garner as much possible information from their received messages as possible. On the other hand, one party might have malicious intrusion and can read the data shared but not necessarily write/change the protocol.

## 3. Fully Homomorphic Encryption and Deep Learning

As a necessary prerequisite, there is some prior understanding about FHE that is necessary but not broadly well-known when applied to deep neural network graphs that are often seen in the field of deep learning. We would like to highlight those here to make it clear in other sections how we overcome these limitations and highlight the advancements we make here. We would also like to note that FHE as a concept is distinct from any specific implementation scheme as we have previously eluded to. In our case, the scheme we use is the (CKKS) scheme as previously stated and described, however, with some further applications as described below.

- Two cyphertexts that operate together must be identical containers: same scheme, same size, same number of primes into their swapping chain, and they must originate from the same private key.
- Additions double the noise of a cyphertext, whereas a multiplication exponentially increases the noise, which means to reduce the noise, we must consume an element in our swapping chain to reduce the noise again. Since multiplication is much noisier than addition, we tend to only swap after multiplication.
- Abelian compatible operations are the only operations that can occur on an FHE cyphertext. This means addition and multiplication. There are methods to model division and subtraction, but these operations are impossible under FHE, thus the need to create new methods and algorithms.
- Cyphertexts size and number of primes in the swapping chain are related. The bigger the cyphertext, the more primes it contains for swapping. However, the bigger the cyphertext, the longer the computation takes. Thus, we want the smallest possible cyphertext that has enough primes to complete the set amount of computations.
- Cyphertexts of a larger size also contain more slots. These slots are what are used to store our message or input plaintext data. Thus, we must also consider that to store a certain number of features, we must have a certain-sized cyphertext. The CKKS scheme has half the number of slots compared to other schemes for the same size since it models pre- and post-point fixed precision.
- Once the swapping chain has been consumed, a very expensive operation called bootstrapping is necessary to refresh the cyphertext and regenerate the swapping chain to continue to conduct noise-expensive operations.
- If the cyphertext is too noisy at the point of decryption, it will lose precision, or if even more noise is present, the decrypted message/data will become garbled and incorrect.

All of these points must be considered in the implementation of FHE compatible neural networks, and this is the primary reason why most existing work in the deep learning field is unfit for use under FHE, including existing deep learning libraries.

We would also like to highlight that as a consequence there is little work in the domain of FHE deep learning with which to compare and draw techniques from.

## 4. Materials and Methods

To enable this research, it was necessary to create our own Python-based FHE-compatible deep learning library because there is still a significant lack of compatibility between existing deep learning libraries and existing FHE libraries. While it may be possible to create some form of interface or bridge, this left much to be desired in terms of usability and flexibility to explore different research avenues such as various FHE backends. As a consequence, we created a NumPy API focused library, where the inputs to the neural networks need only conform to the basic NumPy custom containers specification, allowing the objects passed in to handle their own nature. This means any NumPy conforming object can be used in our networks. This includes NumPy itself (for pure plaintexts) or in this case, arbitrary FHE objects. Our research here focuses on CPU computations as compatibility with existing CUDA implementations is currently infeasible due to compatibility, which means conducting FHE over GPUs would be extremely difficult at this time. Encrypted deep learning accelerated by GPUs is an area we seek to explore in the future. For the rest of this paper, however, all operations are conducted on CPUs. Our entire source code for our library Python-FHEz is available online along with the respective documentation [28]. We use the MS-SEAL C++ library bound to Python using community pybind11 bindings to provide us with the necessary FHE primitives which we then wrap in the NumPy custom container specification for the aforementioned reasons [35].

Furthermore, in this section, we outline the specific implementation, techniques, equations, and methods used to exemplify EDLaaS, using both an open dataset and a preview of a more real-world/complicated but proprietary data scenario. We do this to enable some comparisons to be drawn and to introduce a new way of solving problems encountered in the agri-food industry:

- We chose to use Fashion-MNIST, consisting of a training set of 60,000 examples and a test set of 10,000 examples as our classification example as it is a drop in replacement for the MNIST dataset, while being more complex but still familiar to most.
- We also chose to use an agri-food but proprietary dataset to exemplify a different kind of regression network and how FHE might play a role in this sensitive industry where data sharing/availability is scarce due to a barrier ofn concerns over competition, which FHE might help reduce [36]. Agri-food is also a key industry which has had a troubled few years due to climate change bringing hotter/record-breaking summers, while also being effected by both coronavirus and Brexit shortages in staffing and thus supplying. In addition, it has been established that data sharing is a hindering factor that prevents machine learning technologies from being adopted at scale [37], but some work has already been undertaken around using federated learning to alleviate some of these issues [8].

**Figure 3:**
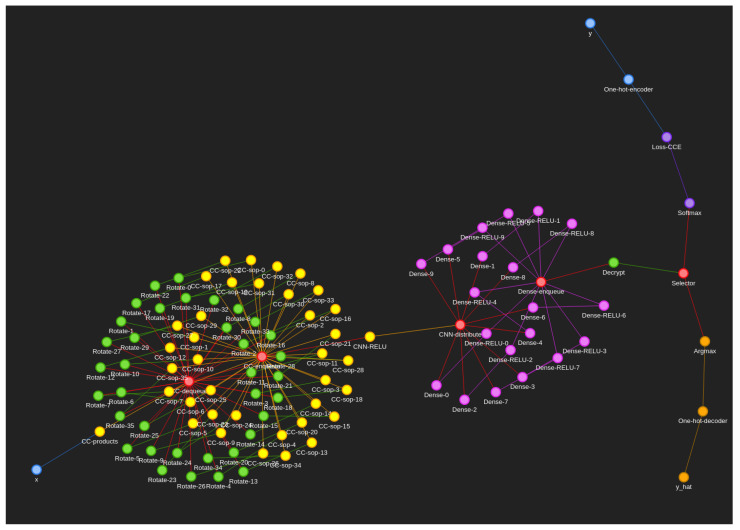
Fashion-MNIST computational graph we call “sphira”, showing the colour-coded graph and the respective nodes used to train/compute Fashion-MNIST using our neuronal firing algorithm. Blue represents the input and input transformation circuit that deals with passing the signals into the neural network in a way it is expecting them. Yellow represents the convolutional neural network components where one filter neuron passes multiple output cyphertexts to a plethora of summing nodes. Pink represents the fully connected dense layer for each class. Purple represents the loss calculation circuit necessary for backpropagation. Orange represents the output/prediction circuit. Red represents the generic glue operations necessary to bind components together. Green represents the encryption specific nodes such as decryption, rotation, and encryption. An interactive version of this graph is available in our source code documentation so that clusters of nodes can be peeled apart for investigating individual nodes and connections [28].

For our neural networks, we used a node-centric, multi-directed graph approach where:

- Each node represents some computation object, usually a neuron.
- Each edge represents the movement of data between neurons/computation objects.
- Each node can accept many inputs that are stacked on top of each other in the same order as the edges unless there is a single input edge where it is instead mapped to the input of the neuron.
- Each edge can only connect two nodes directed from the first to the second node. Parallel edges are possible and are treated as completely separate edges with no special handling.
- A node can only be activated/computed once all predecessor edges carry some data.
- All nodes can have several receptors, that is to say different functions that can be pointed to by the edges, in particular forward and backward receptors for calculating the forward neural network pass and gradients using the chain rule in the backward pass.
- Nodes return either an iterable to be equally broadcast to all successor edges or a generator to generate independent results for each successor edge.
- The weight of each edge corresponds to the computational depth of the directed-to-node. These weights are not used to optimise the path since the majority of nodes must be activated to achieve some desired output, but instead these weights are used to find the longest path between key rotations to determine the minimum required encryption parameters to traverse from one rotation to the next.
- Self-loop edges are not treated differently, instead relying on the configuration of the node itself to consider termination of the loop.
- A single activation pass of the graph may have multiple input and multiple output nodes/neurons, such as in the two blue regions in the sphira graph (Figure 3).

We conduct our study from the node perspective as we find this to be conceptually clearer and follows our own mental abstractions of how neural networks operate. This makes it easier for us to conceptualise, implement, and communicate our neural networks, in particular visually.

To activate our neural network graph, we used our own neuronal-firing algorithm (Algorithms 1–5), since we could not find better existing algorithms that would be suitable for firing of encrypted neuron graphs, while offering us the flexibility to adapt to changing our research.

**Algorithm 1 ** Neuronal-Firing, our exhaustive neuron stimulating, depth first, blocking, node-centric, graph/neuron stimulation function.**Require:** *g*: Neural network multi-directed computational graph**Require:** *n*: Vector of neurons/computational nodes for sequential stimulation**Require:** *s*: Vector of signals to be induced in the corresponding neuron**Require:** *r*: Vector of receptors to call on respective node**Ensure:** $g^{\prime }$: Stimulated neural network/modified computational graph    **for**
i←0tolength(*n*) **do**          signal_carrier(g,n[i],r[i],s[i])


**Algorithm 2 ** Neuronal firing signal carrier; propagate a single signal thought for all possible nodes in the neural network graph recursively based on its position.**function**signal_carrier(g,n,r,bootstrap)    s←get_inbound_signal(g,n,r,bootstrap)    **if** s=None **then**        **return** None    s←apply_signal(g,n,r,s)    **if** s=None **then**        **return** None    set_outbound_signals(g,n,r,s)
**for all**

successorsing.node(n).successors()

**do**
    signal_carrier(g,n,r,None)


**Algorithm 3 **Calculate accumulated inbound signal from edges.**function**get_inbound_signal(*g*, *n*, *r*, bootstrap)    **if** bootstrap≠None **then**        **return** bootstrap    s←[]    **for all** edgesing.in_edge(n) **do**        s.append(edge.signal(r))    **if** length(s)=1 **then**        **return** s[0]    **return** s


**Algorithm 4 ** Activate current node using the accumulated signal and get outbound signal.**function**apply_signal(*g*, *n*, *r*, *s*)    **if** s=None **then**        **return** None    s←g.nodes(n).receptor(r,s)    **return** *s*

**Algorithm 5 **Set outbound edges with activation signal.**function**set_outbound_signals(*g*, *n*, *r*, *s*)    **if** s=None **then**        **return** None    **for all** edgesing.out_edges(n) **do**        **if** isinstance(s,generator) **then**           edge.signal(r)←next(s)        **else**           edge.signal(r)←s


### 4.1. FHE Parameterisation

Our automatic FHE parameterisation approach is similar to that of MS-EVA [11], where we use (in our case our existing neural network) graphs to represent the computation the cyphertexts will experience. This allows us to automatically generate the smallest secure cyphertext possible that meets the requirements of the proceeding computational circuit. How we differ, however, is that since we are using neural network neurons instead of atomic (addition, multiplication, etc.) operations, there are fewer nodes and edges and thus less overhead necessary for both the graph and any intermediate storage along edges. This is because we can block optimise at a higher level than would be possible if purely considering individual atomic operations. Moreover, our neural network graphs are multi-directed graphs (MDGs) as opposed to directed acyclic graphs (DAGs) which means we can model more complex operations involving more than two inputs. This affords us the ability to model the complex relationships in neural networks much like standard deep learning libraries.

In our abstraction, automatic FHE parameterisation becomes a variation of the travelling salesman problem, but instead of finding the shortest path, we need to find the longest possible path, or more specifically the highest computational depth experienced by the cyphertext between sources and sinks. However, even in our abstraction, we must still conform to the constraints of CKKS, i.e., interacting cyphertexts must match in cyphertext scales and must be originating from the same private key which means other adjoining paths must be considered where they intersect. A key distinction compared to MS-EVA’s approach is that our graphs are interpreted instead of being compiled to some intermediate representation. Our cyphertext objects are also not raw and are instead part of larger NumPy-API compatible objects that interpret invocations. These meta-objects are also responsible for the decision making of both relinearisation and re-scaling, taking that complexity away from the implementation of encrypted deep learning. An example of this rescaling interpretation is when two cyphertexts are multiplied, the meta-object is responsible for ensuring both cyphertexts match, i.e., swapping down the modulus chain to equal scales depending on which of the two cyphertexts is higher up the modulus switching chain. Similarly, an example of relinearisation is when two of our meta-objects are multiplied, the computing member (usually the first meta-object in sequence) automatically relinearises the new meta-object before passing the new meta-result back. This means we offload re-scaling and relinearisation, and it is not necessary to plan for these two operations. Instead, we need only calculate the longest paths and the "groups" of cyphertexts. Here, groups of cyphertexts means cyphertexts that interact and must then share encryption parameters.

In short, the minimum necessary information we need to derive from the graph using our algorithms (Algorithms 6 and 7) is:

- Which cyphertexts interact at which nodes;
- Thus, which nodes belong to which group;
- What is the maximum computational depth of each group necessary to go from one (type of concern) source to another (type of concern) sink/rotation.

**Algorithm 6 ** Automatic FHE parameterisation by source and cost discovery over multi-directed graphs.**function**autoHE(*g*, *n*, concern)    **for** iinn**do**                        ▹ Label graph sources and costs        autoHE_discover(g,i,i,concern,0)    r←tuple(dictionary(),list())                  ▹ Group representation    **for** iinn**do**                      ▹ Assign + merge groups from labels        **if** r[0].get(i)isNone **then**           r[0][i]←len(r[1])           r[1].append(0)        **for** jing.nodes() **do**           src←j[1][″sources″]           **if** iinsrc **then**               **for** kinsrc **do**                   r[0][k]←r[0][i]                   **if** src[k]>r[1][r[0][i]] **then**                       r[1][r[0][i]]=src[k]
    **return**
*r*

**Algorithm 7 ** Recursive FHE parameterisation source and cost discovery over multi- directed graphs.**function**autoHE_discover(*g*, *n*, *s*, concern, *c*)    d←g.nodes().get(n)    **if** d.get(″sources″)=None **then**        d[″sources″]←dict()    **if** s≠n **then**        **if** d[″sources″].get(s)=None **then**           d[″sources″][s]←c        **else if** d[″sources″].get(s)<c **then**           d[″sources″][s]←c    **if** isinstance(d[″node″],concern) **then**        autoHE_discover(g,n,n,concern,0)    **else**        **for** iing.successors(n) **do**           nxt←c+g.nodes()[i][″node″].cost()           autoHE_discover(g,i,s,concern,nxt)


**Figure 4:**
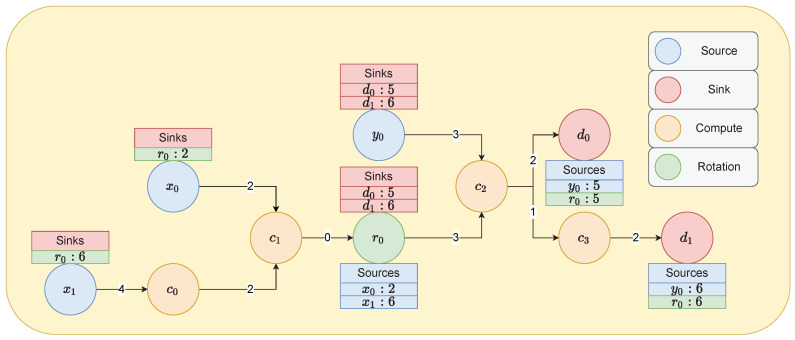
Example automatic FHE parameterisation problem over a multi-directed graph. Sources are where data become a cyphertext. Sinks are where cyphertexts become plaintexts. Computation nodes are generic nodes that represent some operation that can be applied to both cyphertexts and plaintexts. Explicit rotation nodes are where cyphertext keys are rotated, either to refresh them, or to change the form of the cyphertext, potentially into multiple smaller cyphertexts. Please note this does not necessarily follow the colour coding of our other automatically generated graphs [28].

Each of our nodes must be labelled with its computational depth so that the highest-cost traversal can take place. This may need to occur multiple times in a single graph, depending on the number of sources and sinks in said graph. Take for instance $x_{0}$, and $x_{1}$ in the dummy network depicted in Figure 4. The cyphertexts $x_{0}$ and $x_{1}$ passed in must be able to reach the end of both paths leading to $r_{0}$ the very next sink/rotation. To achieve, this they must be interoperable with each other at the point at which they meet. This means they must have matching scales and encryption parameters and must originate from the same private key. However, consider that $x_{1}$ experiences computations $c_{0}$ and $c_{1}$, whereas $x_{0}$ only experiences $c_{1}$. Each computation changes the scale, and thus necessarily their remaining primes in the modulus switching chain which would make them inoperable if not for our specialised logic in the meta-object to match them automatically. For instance, spatial and temporal data in the case of multi-modal datasets (of which Fashion-MNIST is not) would have multiple inputs that require matching. Since decisions on relinearisation and rescaling are left to the meta-object, the only information we need to ordain from the graph is the computational depth and co-dependency of parameters. This can then be used to associate parameters together and select the minimum viable polynomial modulus degree.

In our node centric view of the graphs, we say an edge from node A to B has the cost associated with B. This algorithm should be able to handle multiple cyphertext ingress nodes (*x*, *y*, etc.), multiple cyphertext egress nodes ($\hat{y}$, and any others), and key rotation stages in between that will also need to be parameterised along the way. Our proposed algorithm can be seen in Algorithm 6. The output of this algorithm is a tuple representation of the graphs parameterisation groups. We will know which nodes need to share parameters and what the highest cost of that parameter group is. If we combine this graph parameter representation and some basic logic, we can tune/parameterise automatically. This will of course vary for each implementation of FHE, from CKKS to BFV, for example, requiring different parameters. The difference in parameterisation is why we separate out this final step so that custom functions can be injected.

The FHE parameters we deal with here, primarily geared toward the MS-SEAL CKKS backend, are:

- Scale: computational scale/fixed point precision;
- Polynomial modulus degree: polynomial degree with which to encode the plaintext message; this dictates the number of available slots and the available number total bits which the coefficient modulus chain can contain;
- Coefficient modulus chain: a list of byte sizes with which to switch down the modulus chain; this dictates the computational depth available before bootstrapping or key rotation is necessary.

However, the information we derive from the graph is generic and can be broadly adapted to generate parameters for other schemes also.

We use the default 128-bit security level of MS-SEAL just as MS-EVA [11], being the most similar existing framework. This security level is broadly considered reasonably secure [11,29,30] and matches our threat model of honest but curious.

Lastly, now that we have calculated the groups, the cost of the groups, and the associated nodes that belong to which groups, we can use a rough heuristic (Algorithm 8) to estimate the necessary FHE parameters to accompany these groups. This heuristic can be tuned and overridden for other FHE schemes to more tightly parameterise if necessary.
**Algorithm 8 ** Heuristicallyparameterise RNS-CKKS scheme using expected cost of computation.**Require:** *c*: Integer maximal cost of this cyphertext group.**Require:** *s*: Integer scale power, the scale of the cyphertext. Default: s=40. We advise not to go below 30 due to noise accumulation and lack of prime availability.**Require:** *p*: Float special prime multiplier, the multiplier that dictates the scale-stabilised special primes in the coefficient modulus chain. Default: p=1.5**Ensure:** parms: MS-SEAL RNS-CKKS parameter dictionary/map.  **function**
parameterise(*c*, *s*, *p*)        parms←dict()        parms[″scheme″]←2            ▹ 2 is CKKS in MS-SEAL        parms[″scale″]←pow(2,s)              ▹ scale power        m←[sforiinrange(c+2)]        m[0]←int(m[0]∗p)             ▹ Mult first special prime        m[−1]←int(m[−1]∗p)            ▹ Mult last special prime        b←27        **while** b<sum(m) **do**            b←b∗2        parms[″poly_modulus_degree″]←int(1024∗(b/27))        **return** parms

### 4.2. Open Data Fashion-MNIST

**Figure 5:**
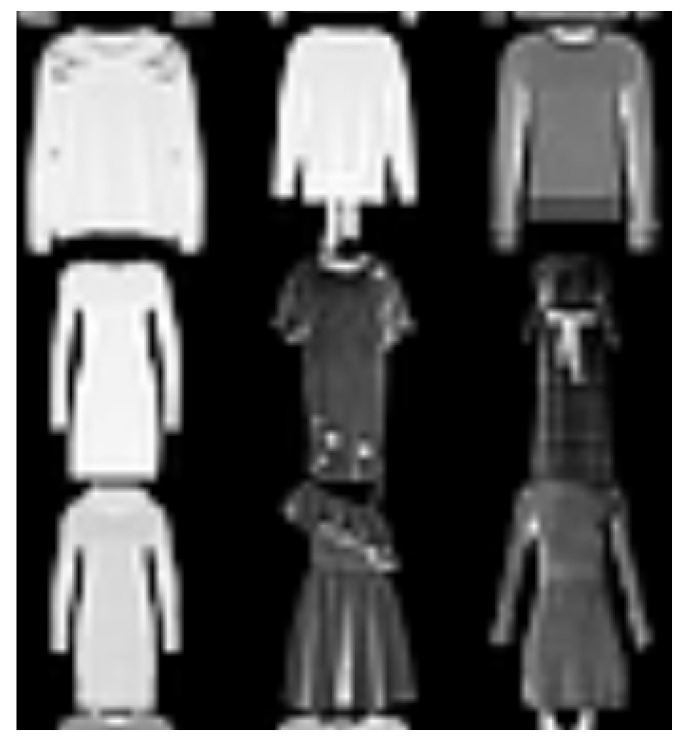
Fashion-MNIST sample showing examples of data such as: boots, bags, jumpers, and trousers [38].

In this section, we describe our openly available Jupyter implementation [28] of an FHE-compatible CNN operating on the open dataset called Fashion-MNIST as can be seen in Figure 5. This dataset contains in total 70,000 images, 60,000 for training and 10,000 for testing. This dataset contains images of certain items of clothing, constituting 10 classes. Each image is a mere 28 × 28 × 1 pixels. The full implementation can be found in the examples of our source code repository [28].

We chose Fashion-MNIST as it is a drop-in replacement for MNIST while also being a somewhat more difficult problem than standard MNIST. Coincidentally, since MNIST and thus Fashion-MNIST are both classification rather than regression, they represent an even more difficult scenario for encrypted deep learning since they do not provide one continuous/regressed output. Therefore, the computational circuit becomes more complex/deeper as far as necessary to process these classifications, i.e., the extra dense nodes for each class, and the whole addition of both softmax and categorical cross entropy (CCE) to replace the mean squared error (MSE) loss function in the case of would-be regression networks. This also poses a problem as methods usually used towards classification such as softmax are not compatible with FHE since they include division although some alternative approximations do exist such as those used by Lee [29].

#### 4.2.1. Data Wrangling and Inputs

Fashion-MNIST is largely pre-wrangled especially if you use one of many forks of the data which present each figure classification and image as a one-dimensional feature vector between 0–255 stacked in a CSV file. This means the only two necessary steps towards this data are to normalise between 0–1 and reshape the individual feature vectors back into their original shape of 28 × 28 × 1. The feature vector is encrypted, and the cyphertext is passed in as a signal to node “x” in the sphira network (Figure 3). The figure classification is passed in to node “y” as a separate signal, whereby our neuronal firing algorithm (Algorithm 1) will propagate these signals thereafter.

#### 4.2.2. CNN

**Figure 6:**
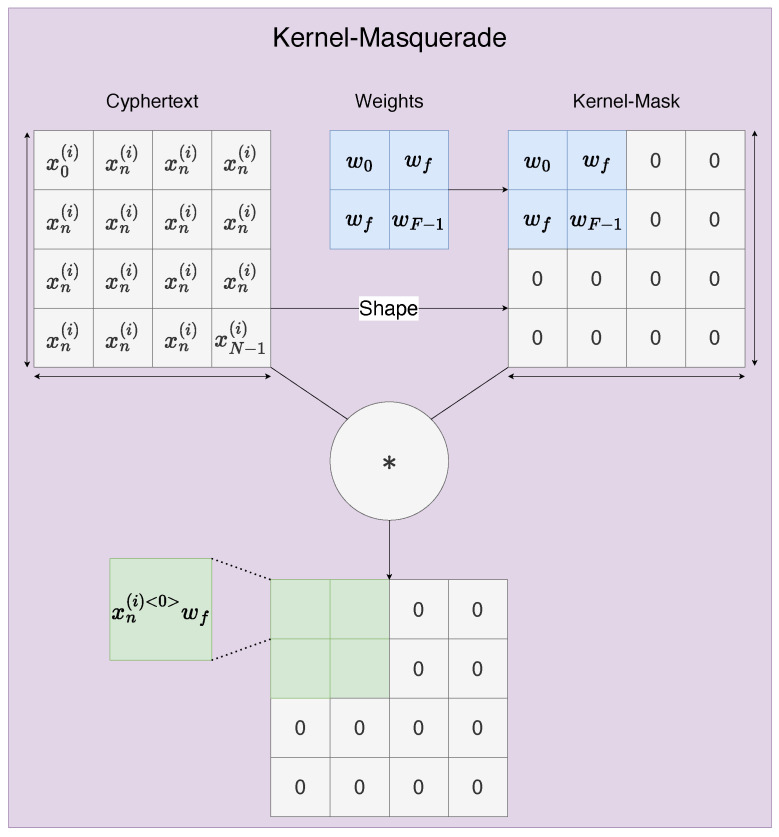
A mergedmask and kernel together to create a single sparse kernel which zeros undesired components in the cyphertexts polynomial of values using Hadmard products. Please see our documentation for closer detail [28]. Symbol ‘∗’ denotes element-wise multiplication.

As our CNN (yellow in Figure 5), we use a biased cross-correlation layer (CC) to calculate the product of a given filter against the input cyphertext. We use an SIMO scheme we call kernel masquerading. Here kernel masquerading means the merging of weights and a respective zero mask into a sparse n-dimensional array such that they become a single operation conducted on the input cyphertext (Figure 6), reducing the computational depth experienced by the input cyphertext to one (multiplication) and allowing for subset operations to be conducted on the cyphertext to selectively choose regions of interest. This is only possible in the plaintext weights strategy, since this allows the weights to be operated on arbitrarily and selectively to reform them into the shape of the input cyphertext and sparsity of the filter/kernel. This is a simple operation of which a two- and three-dimensional variant can be seen in Juvekar’s, and Meftah’s work [30,31]. The main drawback of the kernel masquerade is that if we were to apply a convolutional kernel mask on some cyphertext $\varepsilon (x^{\left(i\right)})$, we would end up with separate modified cyphertexts $\varepsilon (x^{(i)<t>})$ that correspond to different portions of the data. However, we are unable to sum them without a key rotation such that we are summing between different cyphertexts since we cannot fold a cyphertext in on itself. This means we have a choice at this stage. We can either rotate the keys now to reduce complexity or try to save computation time by doing as much processing while the values are encoded in one larger cyphertext which is significantly more efficient from findings in Juvekars work on SISO cyphertexts [31].

Since key rotation would make the outputs normally processable for operations such as summation, we will not address that variant here. Instead, we choose to see how far we can commute this sum to obtain the maximum performance as far fewer cyphertexts. One thing we can and did do in our CNN implementation is to commute the bias forward to be before summation. So, instead of $z=\sum_{i=0}^{N}\left(x_{i}w_{i}\right)+b$, we decompose *b* into the product calculation before summation as $z=\sum_{i=0}^{N}\left(x_{i}w_{i}+\frac{b}{N}\right)$ since this is equivalent over the full computation of the cyphertext. We could have simplified to just $z=\sum_{i=0}^{N}\left(x_{i}w_{i}+b\right)$ if we calculate the gradient with respect to the bias $\frac{df}{db}$ as $\frac{df}{db}=Nx$ instead of $\frac{df}{db}=x$ such that the neural network is effectively aware of this higher contribution of the bias, and it would be naturally accommodated through the gradient descent process.

From here forward, special logic/considerations need to be made to ensure the output cyphertexts of the biased-cross-correlation are treated as a singular un-summed value. We tried to push this cyphertext through the neural network further, but we had to ensure all further operations were both linear and abelian compatible. Take for instance an encoded non-summed sequence as a cyphertext *x*, $x=\left(1+2+3+4\right)=10_{\mathrm{plntxt}}$. Then, let us try a multiplication 4x=4(1+2+3+4)= (4 × 1 + 4 × 2 +4 × 3 + 4 × 4) = (4 + 8 + 12 + 16) = 4 × 10${}_{\mathrm{plntxt}}=40_{\mathrm{plntxt}}$. Now, let us try a multiplication against itself or another non-summed sequence, for instance a nonlinear $x^{2}=\left(1\;+\;2\;+\;3\;+\;4\right)\left(1\;+\;2\;+\;3\;+\;4\right)=$ (1 × 1 + 1 × 2 + 1 × 3 + 1 × 4 + 2 × 1 + 2 × 2 + 2 × 3 + 2 × 4 + 3 × 1 + 3 × 2 + 3 × 3 + 3 × 4 + 4 × 1 + 4 × 2 + 4 × 3 + 4 × 4) = $10_{\mathrm{plntxt}}^{2}=100_{\mathrm{plntxt}}$.

This is a problem since we cannot cross-multiply cyphertexts because we cannot select elements from either cyphertext. If we were to attempt to multiply this cyphertext with itself, it would calculate the element-wise product of the two. The best we could do if we did want to compute this, would be to conduct a key rotation to expose the elements we desired as separate cyphertext. However, if we are going to do that, we would be just as well served by just rotating to sum then passing it through the element-wise product as normal. It is possible to commute the sum further if we use linear approximations of our activation function. For example, if we take sigmoid (Equation (1)) and its approximation Equation (2), then if we ensure our products will always be between 0-1 through a modified version of batch norm ([30]). We could then safely use only the linear component of the sigmoid approximation (Equation (2)) $\sigma \left(x\right)\approx \sigma_{a}\left(x\right)=0.5+0.197x$ since it would still closely follow in the −1 to 1 range and loses approximation beyond this range instead of the usual −5 to 5 range the full approximation affords and would also cut down the computational cost. For ourselves, we choose to key rotate to encrypt the elements to be summed until such a time as we have fully fleshed out a fully commuted sum alternative.

(1) $$\begin{array}{c} \sigma \left(x\right)=\frac{1}{1+e^{-x}} \end{array}$$

(2) $$\begin{array}{c} \sigma \left(x\right)\approx \sigma_{a}\left(x\right)=0.5+0.197x+-0.004x^{3} \end{array}$$

For our cross-correlation activation function, we use ReLU (Equation (3)) approximation (Equation (4)) and the derivative of this approximation for backward propagation (Equation (5)) as its own separate node to allow them to be decoupled and easily swapped out with new or improved variants so interjection with batch norm is readily variable without having to rewrite existing nodes.

(3) $$\begin{array}{c} R(x)=\max(0,x) \end{array}$$

(4) $$\begin{array}{c} R\left(x\right)\approx R_{a}\left(x\right)=\frac{4}{3\pi q}x^{2}+\frac{1}{2}x+\frac{q}{3\pi } \end{array}$$

(5) $$\begin{array}{c} \frac{dR(x)}{dx}\approx \frac{dR_{a}\left(x\right)}{dx}=\frac{8}{3\pi q}x+\frac{1}{2} \end{array}$$

#### 4.2.3. Dense/ANN

**Figure 7:**
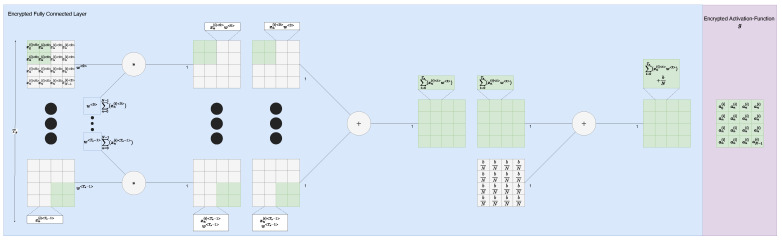
Encryptedvariant of an ANN/dense neural network, usually used in our case to merge divergent times/branches/filters back together into a single output. Symbol ‘∗’ denotes element-wise multiplication. Please see our documentation for closer detail [28].

For each of our classes, we have a dense fully connected neuron (Figure 7, red in Figure 5) to interpret the activation vector of the biased cross-correlation and activation combination/CNN. Thus, our dense layer is comprised of 10 ANN nodes. There is nothing of any note in this layer other than it must accept multiple cyphertexts that are added together/summed across the first axis; otherwise, it behaves almost the same as a standard neuron as depicted in the figure. However, careful attention should be paid to broadcasting such that the gradient is still correct, and we do not attain an exploded result that could fall outside of approximation golden zones such as sigmoids −5 to 5.

We accompany each neuron with its own ReLU approximation node before passing the activations on to the different forward evaluation circuits for loss calculation and prediction output.

#### 4.2.4. Prediction

Argmax is an effective and quick computation of the highest value in a vector. Since the ANN layer outputs a vector of 10 values, 1 for each class, the Argmax function serves to take the highest activation and turn it into a 1-hot-encoded representation of the predicted class. This can be passed into a one-hot-decoder to attain the predicted class $\hat{y}$. However since argmax relies on the context to find the max, it is necessary to conduct this operation in plaintext on the client side to effectively pick from this 10 element vector. There is no backpropagation from this branch; it is purely an output branch for providing predictions to the data owner. These stages are pink in Figure 3.

#### 4.2.5. Loss

The loss calculation stage is represented by purple in Figure 3. Argmax is not an effective function for the purposes of backpropagation of the loss since only one of the ten input ANN neurons would receive all of the gradient multiplied by one, which does not give the majority of the network much information to update the weights from any single given example. Thus, as per norm we used a softmax layer instead which better distributes the gradient between not only what neuron was responsible for positive activation, but also the others that should not be activating.

The softmax ensures that all output values summed together equal one and that they are effective predicted probabilities of the network that a certain class is what was given in the input. We use a standard categorical cross-entropy (CCE) function to calculate the loss and subsequently the derivative with respect to each of the 10 classes to pass back to the softmax and hence the ANN layer.

The CCE function also receives input/stimulation from a one-hot-encoder that encodes the ground-truth *y* value or the actual class that the input *x* corresponds to for the purposes of loss calculation.

(6) $$\begin{array}{c} \sigma {\left(\vec{a}\right)}_{i}=\frac{e^{a_{i}}}{\sum_{j=1}^{K}e^{a_{j}}} \end{array}$$

It should also be noted that the loss circuit (pink) requires decryption since both softmax and CCE are not FHE-compatible operations. There have been proposed ways to allow for softmax to be computed with cyphertexts by Lee [29]. However, we were unable to create a working FHE-compatible softmax from what information was available and would require bootstrapping 22 times, and it would still need to be unencrypted for the CCE calculation. Given this data, using the sphira (3) network we garnered the following results.

### 4.3. Strawberry Yield Data

Unfortunately, we do not have permission to publicise the specific data used in this section. As such, we shall preview this application and seek to further elaborate and develop the techniques used here in-depth in future papers. We will only touch briefly here of this data as a means to show how FHE can be used in real-world problems effectively, and for a different kind of problem: regression instead of classification.

These data are geared towards yield prediction, often weeks in advance. The prediction horizon is typically between one to three weeks ahead of the strawberry fruit becoming ripe. This allows time for logistical constraints, such as price negotiation, and picker/staff scheduling. Thus, performance of these predictive models is critically important to ensure all the fruit is being accounted for in negotiations with retailers (and thus can be sold) and that there is sufficient manpower at the point of need to gather this produce. Over-predicting can result in insufficient harvests to meet contractual obligations, most likely meaning the yield must be covered by buying other producers’ yields. However, if there is a shortfall of yield from one producer, the factors that lead to that shortfall, such as adverse environmental conditions, are felt by most other producers in the geographic region. This means that usually (in the UK) the yields must be imported from abroad, increasing the price substantially. Conversely, under-prediction of yield results in unsold fruit, which is either sold at a significant discount if possible or destroyed. There is also a clear and significant lack of agriculture data available due to perceived data sensitivity. This affects many forms of agriculture for various reasons. In the soft-fruit industry, this tends to be proprietary genetic varieties and operational specifics such as irrigation nutrition mixtures. However, there is a tendency to distrust and a perceived lack of benefit to data sharing due to no obvious performance outcomes.

Due to a lack of available data, we use historic yield data we gathered in our Riseholme campus polytunnel/tabletop over two years and combine this with environmental data experienced by these strawberries leading up to the point of prediction. The environmental data includes: wind speed, wind direction, temperature, light-intensity, humidity, precipitation, positions of strawberries, yield per row of strawberries, and many more less significant features. This data also include irrigation data such as nutrition, soil moisture, soil temperature, and irrigation status. We normalised one-hot-encoded categorical variables and split the data (80-20) randomly into training and test sets. We then further subdivided the training set into validation sets for model selection purposes.

**Figure 8:**
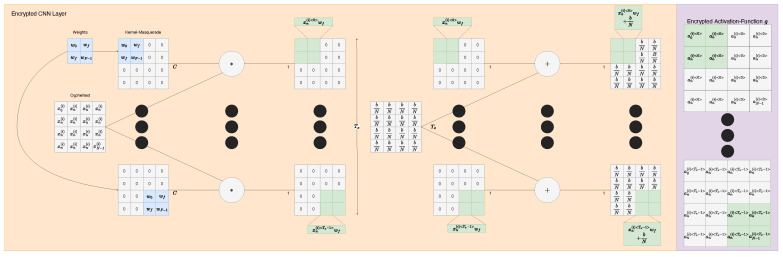
Encrypted convolutional neural network (CNN). This is a particularly unusual implementation since there can be no summing of the filters. Instead, this sum is commuted in the case where the filter operates on an input that is a single cyphertext (i.e., not a composite of multiple cyphertexts). Please see our documentation for closer detail [28].

We applied a 1D/time-series CNN (Equations (2), (7) as depicted in Figure 8) followed by a dense ANN and sigmoid again as depicted in Figure 7) to summarise the feature vector and to predict the output/yield of the strawberries. This prediction will then be based on the environment the strawberries experienced leading up to the point of prediction. Given our data and our feature engineering, we were able to obtain the following outcomes in Table 1.

**Table 1:** Table of predictive results of the constellation network (Figure 9) predicting strawberry yield.

Days Ahead	Mean Absolute Percentage Error (MAPE)
7	8.001
14	14.669
21	22.326

(7) $$\begin{array}{c} a^{(i)<t>}=g\left(\sum_{t=0}^{T_{x}-1}\left(k^{<t>}x^{\left(i\right)}+b/N\right)\right) \end{array}$$

## 5. Results

**Figure 9:**
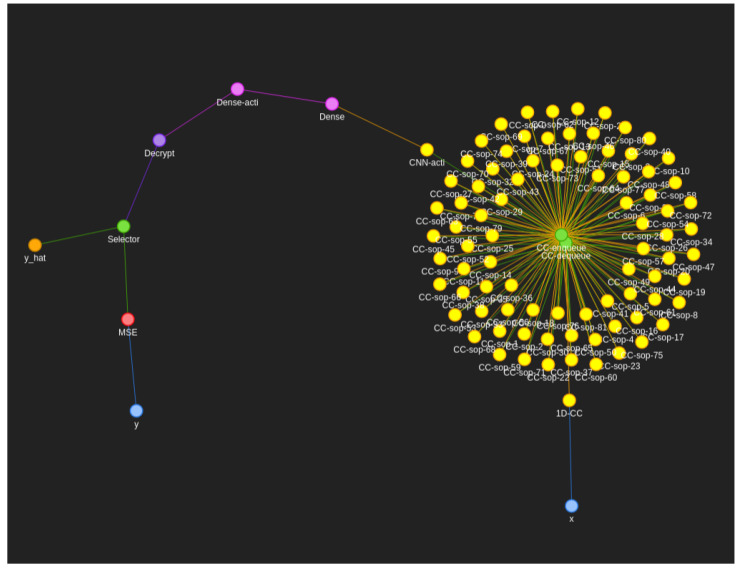
Strawberry yield/regression computational graph we call ‘constellation’, showing the colour coded graph representation and nodes used to train on strawberry yield based on environmental factors. Blue are input/encryption nodes. Yellow are convolutional-related nodes. Green are operational nodes necessary to “glue” the network together. Pink are dense/ANN nodes. Orange is the output prediction node. Red is the loss calculation node. Purple is an FHE-specific node used for decryption of the input data. Please see our documentation for closer detail [28].

**Figure 10:**
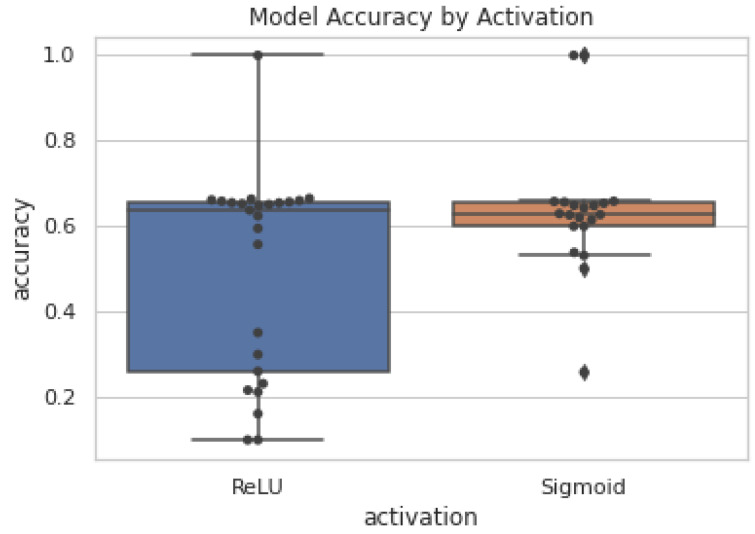
Model performance using different activation functions in the sphira network on the fashion-MNIST dataset. All activations here are their FHE-compatible approximations unless otherwise specified. Each dot is a different network or the same network with a different data type(cyphertext, plaintext) [28].

As can be seen in Figure 10, using the same network sphira (Figure 3) with different approximated activation sigmoid functions (Equation (2)) and ReLU (Equation (4)) dramatically affects the precision of the neural network over multiple training attempts with randomised weights. However, the accuracy of both on average is roughly equal within a few percent. This shows that in our implementation, backpropagating the ReLU approximation may indeed cause some instability more frequently. Sigmoid, in contrast, is a static approximation which may be part of the reason for its greater stability, and thus consistency, in providing randomised weights to the rest of the network. We can see that both the sphira (Figure 3) and constellation (Figure 9) networks can produce acceptable results on the testing set while computing over cyphertexts and plaintexts. Our networks can be seen working in both classification and regression problems, Fashion-MNIST, and strawberry yield prediction, respectively.

**Figure 11:**
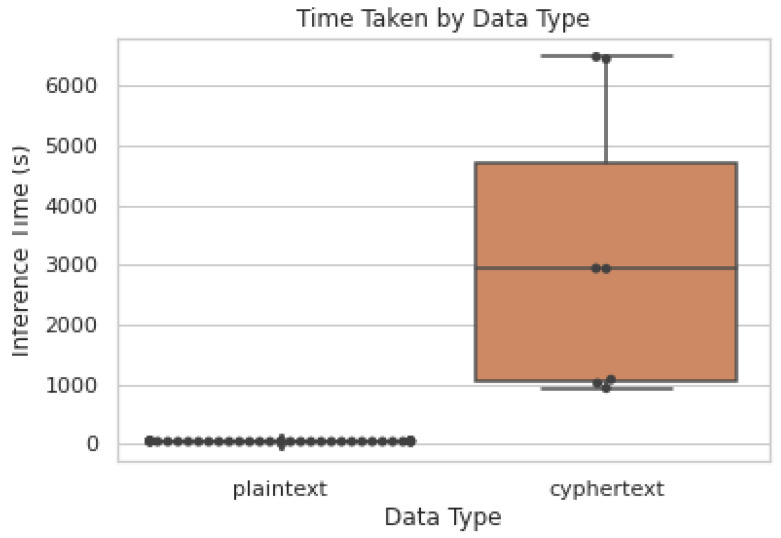
Model inference time by different types. Plaintext types mean where the graph is run using plaintext data. Cyphertext types mean where the graph is run using cyphertext data. Both plaintext and cyphertext data conforms to the same NumPy API, meaning they can be used interchangeably. Each dot is a different network (i.e., differently initialised weights but the same structure) or the same network with a different data type (cyphertext, plaintext) [28].

We find, however, that in our strawberry yield prediction, one of the weaknesses of our approach was to completely randomise the sequences, as some sequences could possibly overlap. This means that the network may have at least some prior experience of the gap between point of prediction and point predicted. An area of possible future expansion is to split the data differently by time and use only environmental data that is distinct in the future. Another area where improvement could be realized is by using smaller but bootstrappable cyphertexts. This may reduce predictive performance of the networks since each bootstrapping operation would incur a noise penalty, but this would significantly improve the speed of computation since we could use smaller cyphertexts that take less time to transverse, transmit, and compute. We can see from Figure 11 that the time taken for computing plaintexts is relatively small, producing results rapidly. The same network, however, provided cyphertexts yields near equivalent, but significantly slower, results. Not only are cyphertexts more time-intensive, but they are also significantly more space-intensive. We could see during cyphertext inference anywhere from 72-80GB of RAM usage, meaning this is certainly not plausible on low-specification machines. We could see significant gains in computational performance if we added more rotation nodes to refresh the cyphertext more frequently to limit the number of levels it would contain, and thus the size of the cyphertext. However, in our case we wanted to reduce the number of rotations as in practical applications this would result in more transmissions from client–server which itself can be an expensive operation. This is a good example of why bootstrapping, while incurring a high cost itself, could save computational time and space in the future, when it is more widely available.

The absolute performance of the two models in Figure 10 and Table 1 is acceptable despite being fairly shallow models compared to those used in many normal deep learning models. Our absolute performance is probably quite limited by the shallowness of these models in that the model may not be complex enough to properly model some of these problems. In particular, there are a plethora of standard models that achieve 90% accuracy or greater, many of which use 2–3 convolutional layers, with batch normalisation, and max-pooling. Clearly, we cannot capture context from a cyphertext making max-pooling impossible. However, we can and do use strides as a way to reduce the dimensionality in a similar way that max-pooling does. There have also been proposals for batch normalisation that involve multiplying by small fractions that are occasionally recalculated. However, this is quite complex and not something we have been able to implement as of yet. This would, however, stabilise the activations between nodes and reduce the likelihood of escaping the dynamically predicted range in the ReLU approximation causing the in-precision in Figure 10.

The fact that we can obtain acceptable performance in different scenarios, such as agricultural yield regression and image classification, opens avenues for data sharing. There are two avenues in particular: through encryption and through trust. In our case, we assume a semi-honest threat model, yet we have outlined a way of computation that does not need to reveal any data to the third party. This means if we can provide sufficient predictive performance, then there are few barriers preventing sharing of encrypted data for inference. There is of course the notable exception of any yet unknown vulnerabilities in the underlying FHE scheme with default parameters provided by MS-SEAL. The other avenue of data sharing that FHE fosters is that of trust. Given a track record of reliable data processing in the encrypted form, could lead to an increased awareness of the gains of deep learning applied to various fields. With this greater awareness and track record, it could be surmised that it is more likely that over time the data owners might choose to share data in the perceived sensitivity scenario.

## 6. Discussion and Limitations

Considering the importance around ascertaining privacy when developing new machine learning methodologies, it is paramount that we start scaling up research on privacy-enabled machine learning. This should take place in tandem with showing how real-life problems, e.g., strawberry yield forecasting, can be tackled with such methodologies, which is what this paper has contributed to, results-wise as well (Table 1). Nevertheless, our work on encrypted deep learning has certain limitations we would like to highlight:

FHE Training: In this paper, we laboriously implement, describe, and show how encrypted deep learning inference can be conducted. However, there is little reference to encrypted learning, that is, where a neural network is trained on cyphertexts. This is due to multiple limitations prevalent in the field, such as the lack of FHE compatibility with certain functions, such as loss functions. This is an active area of research which we and the broader research community are actively working on to complete the encrypted learning to inference chain. Another issue with cyphertext training is deciding when we stop training. This is a particularly interesting and challenging problem which we seek to also tackle in the future. Here, however, we work to avoid privacy leaks related to training.

LFHE: As previously mentioned, our work here is over Levelled-FHE, where we create optimised circuits for cyphertexts with discrete scales and primes, which we swap down for each multiplication, a “level”. LFHE is FHE without bootstrapping. Bootstrapping is an expensive operation that refreshes the cyphertexts levels, allowing for an effective limitless depth to computations (albeit with noise), while also helping to keep cyphertexts smaller than their LFHE counterparts. Smaller cyphertexts can be operated on faster, but bootstrapping in small circuits can often outweigh the benefit of using a smaller but bootstrapped cyphertext, due to how expensive of an operation it is. This limitation comes from a lack of bootstrapping support in MS-SEAL. Once bootstrapping is supported, however, existing networks we propose here will still be compatible, assuming appropriate NumPy-container abstractions of FHE will be passed in.

State-of-the-Art Neural Networks: While this work focuses on ANN and CNN neural networks, these are not current state-of-the-art networks for many tasks. In particular, in future we intend to continue to work applying FHE to existing networks, such as transformers, which are SotA in sequence tasks. However, much work remains in mimicking certain functions of transformers in an FHE-compatible manner [12]. We also believe while we could compute privately, we can significantly improve the performance of the predictions themselves with better performing network architectures such as transformers. We could then draw more comprehensive comparisons between encrypted and unencrypted deep learning for yield forecasting and other applications.

## 7. Conclusions and Future Work

In this paper, we have shown how FHE can be automatically parameterised directly from multi-directed graphs for neural networks, using groups and a variation of the travelling salesman problem for costs. It was demonstrated how multi-directed graphs can be used in an FHE-compatible manner with FHE-compatible nodes to facilitate encrypted deep learning. We have also evaluated a recent ReLU approximation (with additionally backpropagated approximation range), against the sigmoid activation function, finding it slightly less accurate but much less precise due to instabilities in the weight initialisation. The proposed encrypted deep learning procedures were utilised in both classification and regression problems. For the former, we used an open dataset Fashion-MNIST with open-source reproducible code examples to aid reproduction and experimentation. For the latter, We demonstrated how our methods can be used in an real world (sensitive) problem predicting strawberry yield, paving the way to introduce such a technology at scale in the agri-food sector. We believe that our implementation is the most comprehensive encrypted deep learning library currently available, now with automatic FHE parameterisation, traversal, cross-compatible/interoperable NumPy custom-containers, documentation, and expandability for future distributed or GPU accelerated computations with FHE, using the state-of-the-art RNS-CKKS FHE scheme provided by the MS-SEAL backend.

However, there is still much research that needs to be conducted, in particular with FHE and training. Encrypted deep learning is not a solution currently to any problem that relies on very specific data that is very dissimilar to other problems, meaning we cannot transfer some understanding in a private manner. We are still limited by multi/parallel processing. However, in the case of Python-FHEz, we leave the backend open-ended following the NumPy custom container specification such that this gap can be easily retrofitted later, just like Dask and CuPy have for standard NumPy.

Finally, with encrypted deep learning, we can open avenues for data sharing that have previously been untenable in the face of their rightful privacy concerns. The more of the pitfalls of FHE that are solved and the more usable encrypted deep learning becomes, the more likely we are to see it provide some critical predictive service to improve fields such as agriculture and medicine.

## Data Availability

There is partial availability of the datasets used in this paper. The first dataset we use called Fashion-MNIST can be found at the following address https:github.com/zalandoresearch/fashion-mnist. The second strawberry dataset is unfortunately currently not permitted to be shared.

## Abbreviations

The following abbreviations are used in this manuscript:

FHE	Fully Homomorphic Encryption
CKKS	Cheon, Kim, Kim and Song
ReLU	Rectified Linear Unit
CCE	Categorical Cross-Entropy
ANN	Artificual Neural Network
CNN	Convolutional Neural Network
RNN	Recurrent Neural Network
MDG	Multi Directed Graph
DL	Deep Learning
MSE	Mean Squared Error
DAG	Directed Acyclic Graph
CC	Cross Correlation
SIMO	Single Input Multiple Output
